# Environmentally Friendly Production of D(−) Lactic Acid by* Sporolactobacillus nakayamae*: Investigation of Fermentation Parameters and Fed-Batch Strategies

**DOI:** 10.1155/2017/4851612

**Published:** 2017-09-10

**Authors:** Susan Michelz Beitel, Luciana Fontes Coelho, Daiane Cristina Sass, Jonas Contiero

**Affiliations:** ^1^Department of Biochemistry and Microbiology, Institute of Bioscience, São Paulo State University (UNESP), São Paulo, SP, Brazil; ^2^Associate Laboratory IPBEN, UNESP, Av. 24A 1515 CEP, 13506-900, Rio Claro, SP, Brazil

## Abstract

The interest in the production of lactic acid has increased due to its wide range of applications. In the present study, the variables that affect fermentative D(−) lactic acid production were investigated: neutralizing agents, pH, temperature, inoculum percentage, agitation, and concentration of the medium components. An experimental design was applied to determine the optimal concentrations of the medium components and fermentation was studied using different feeding strategies. High production (122.41 g/L) and productivity (3.65 g/L·h) were efficiently achieved by* Sporolactobacillus nakayamae* in 54 h using a multipulse fed-batch technique with an initial medium containing 35 g/L of yeast extract (byproduct of alcohol production), 60 g/L of crystallized sugar, and 7.5 mL/L of salts. The fermentation process was conducted at 35°C and pH 6.0 controlled by NaOH with a 20% volume of inoculum and agitation at 125 rpm. The production of a high optically pure concentration of D(−) lactic acid combined with an environmentally friendly NaOH-based process demonstrates that* S. nakayamae* is a promising strain for D(−) lactic acid production.

## 1. Introduction

Lactic acid production has received greater attention due to its considerable potential in biotechnological applications in a wide range of fields as well as the increasing need for new biomaterials, such as biodegradable, biocompatible, and polylactic products [1–3]. This compound is used in the food, chemical, pharmaceutical, and cosmetic industries, is amenable to several chemical conversions, and is used as a precursor for various chemicals and materials [4].

The two optically active isomeric forms of lactic acid are D(−) and L(+). Lactic acid can be obtained by chemical synthesis, which inevitably leads to the production of a racemic mixture, whereas the pure enantiomers L(+) or D(−) lactic acid can be produced through fermentative processes. The industrial production of L(+) lactic acid has been extensively reported using several substrates, but few studies have addressed the commercial production of D(−) lactic acid [5].

Lactic acid is becoming important as an intermediate feedstock used as a monomer for the synthesis of biodegradable polymers [6]. Lactic acid polymer [polylactic acid (PLA)] has three different forms: poly-L-lactic acid, poly-D-lactic acid, and poly-DL-lactic acid [7]. PLA is used in the field of biomedicine in the form of implants, sutures, bone fixation material, and microsphere drug delivery systems due to its excellent biocompatibility and biodegradability [8, 9].

For the production of lactic acid, the most important carbohydrates are the disaccharides lactose, maltose, and sucrose [10]. Sucrose enters cells through a specific permease system and is split into glucose and fructose [11]. Since PLA manufacturers require large amount of lactic acid at a relatively low cost, the determination of inexpensive raw materials is important to the feasibility of the microbial production of lactic acid [12]. However, the raw materials used as the carbon source must undergo hydrolysis in order to make the sugars assimilable to the bacteria [13]. Thus, pure sugars facilitate downstream processing, whereas inexpensive raw materials as alternative substrates result in an increase in the downstream processing cost due to their relatively high amount of impurities [14].

The fermentation rate depends mainly on pH, temperature, agitation, initial substrate concentration, and the concentration of nitrogenous nutrients [15]. Batch fermentation is widely used in lactic acid production. However, the long fermentation times required with this technique results in low productivity as well as low cell concentrations. The inhibitory effects of the initial substrate concentration on production are also considered a major drawback of this fermentation method. To solve such problems, fed-batch fermentation has been investigated to improve the production, productivity, and yield of lactic acid [3].

Lactic acid is produced by several microorganisms, especially lactic acid bacteria, including genetically modified strains, and some fungi [15–18]. The species* Sporolactobacillus nakayamae* belongs to the family Sporolactobacillaceae and is an endospore-forming, microaerophilic, mesophilic, homofermentative, and Gram-positive bacterium that exclusively produces D(−) lactic acid [19]. The aims of the present study were to investigate the influence of the concentration of culture medium components as well the effects of temperature, pH, inoculum percentage, agitation conditions, and pH controlling agents on D(−) lactic acid production by* S. nakayamae*. Fed-batch strategies were also studied to increase production and productivity levels of D(−) lactic acid.

## 2. Materials and Methods

### 2.1. Bacterium Strain and Culture Medium

The producer strain was the previously identified* as Sporolactobacillus nakayamae* by Macrogen (Korea). A glucose, yeast extract, and peptone (GYP) medium containing 20% (v/v) glycerol was used for the maintenance of the strains at −80°C. The cells were propagated three times in GYP broth before use and incubated at 35 ± 1°C under stationary conditions until the optical density (OD_600_) reached 2.5 (about 24 h).

### 2.2. Optimization of Fermentation Media

#### 2.2.1. Response Surface Methodology

The response surface methodology (RSM) is a set of experimental strategies, mathematical methods, and statistical inferences that enable efficient empirical exploration in a reduced number of experiments [20]. To evaluate the influence of crystallized sugar, yeast extract, and the salts solution (4% MgSO_4_·7H_2_O, 0.16% MnSO_4_·4H_2_O, 0.2% FeSO_4_·7H_2_O, and 0.2% NaCl) on lactic acid production by* S. nakayamae*, the RSM was used with three replicates at the center point, totaling 17 experiments. The medium used for fermentation was formulated based on the previously used GYP medium. Crystallized sugar is a commercial table sugar found at an affordable price in Brazil. The yeast extract was from Zilor (a major ethanol-producing company in Brazil) and is a byproduct of ethanol fermentation, which is processed and available at a very low cost. The levels used for encoding the independent variables are shown in Table 1.

Fermentation runs were carried out in 125 mL Erlenmeyer flasks with an inoculum concentration of 10%, initial pH at 6.0, and 5% CaCO_3_ used as the pH neutralizer. The flasks were incubated for 48 hours at 35°C under stationary conditions. D(−) lactic acid and residual sugar were quantified at the end of fermentation. To validate the optimization of the medium composition, five repetitions were carried out using the optimized conditions (120 g/L of crystallized sugar, 35 g/L of yeast extract, and 7.5 mL/L of GYP salt solution) to confirm the results of the response surface analysis. The results were expressed as mean values. The experimental design was determined using the Statistica 7 software program (StatSoft, Tulsa, USA), which was also used for the analysis of the results.

### 2.3. Effect of Culture Conditions on Lactic Acid Production

Variations of different process parameters (pH, pH controlling agent, temperature, agitation, and inoculum volume) were tested to optimize D(−) lactic acid production. Fermentation was conducted in a bioreactor (Infors HT Multifors 2) with a working volume of 300 mL of modified GYP medium (35 g/L of yeast extract, 120 g/L of crystallized sugar, and 7.5 mL/L of GYP salts solution) and 10% (v/v) of inoculum (except in tests on the influence of inoculum size on lactic acid production). The effect of pH on lactic acid production was evaluated from 5.0 to 7.0. The influence of the pH controlling agent was evaluated throughout fermentation by the automatic addition of controlling agents. The influence of temperature was determined from 31 to 39°C and the influence of agitation was evaluated from 0 to 150 rpm. The production medium was inoculated with different inoculum levels (5 to 25% v/v). Fermentation was stopped after 48 hours. Samples were withdrawn periodically from the bioreactor to determine the concentrations of sucrose and D(−) lactic acid. The final fermentation volume was considered for the calculation of lactic acid normalization (production g/L × final volume mL/initial volume mL).

### 2.4. Fed-Batch Fermentation Strategies

Fed-batch fermentation was performed in a 750 mL bioreactor (Infors HT Multifors 2) with a working volume of 300 ml of optimized medium containing 35 g/L of yeast extract, 7.5 mL/L of salts solution, and various amounts of crystallized sugar, as required. Fermentation was run at 35°C and 125 rpm with an inoculum volume of 20% (v/v). NaOH was added automatically to control the pH at 6.0. The feed solution contained 730 g/L of crystallized sugar and 1% of yeast extract. In fed-batch fermentation, the feed solution was pumped into the fermenter using a peristaltic pump coupled to a computer. Samples were withdrawn at established intervals for the determination of lactic acid production, sugar consumption, and cell growth. Batch fermentation was conducted with an initial crystallized sugar concentration of 120 g/L and NaOH was added throughout the process to control the pH.

Different feeding strategies were tested to enhance lactic acid production. In the single-pulse fed-batch fermentation, the initial concentration of crystallized sugar was 120 g/L. The feed solution was supplied once when the residual sugar concentration decreased to 60 g/L (at 18 hours) to bring the concentration up to approximately the initial value. Multipulse fed-batch fermentation was conducted with an initial crystallized sugar concentration of 60 g/L, followed by several additions of the feed solution when the residual sugar concentration was below 40 g/L. In the constant fed-batch fermentation, the feed solution was added when the residual sugar concentration decreased from the initial 120 g/L to 60 g/L (at 18 hours) and pumped at a rate of 3 ml/h until 30 hours. In exponential fed-batch fermentation using the Irirs 6 software, a control strategy developed by Nor et al. (2001) [21] was used, in which the feed rate (*F*) was determined by B, which takes into account a mass balance, assuming a constant cell yield on substrate as well as a constant maintenance coefficient throughout the fermentation.

(B) $$\begin{array}{c} F=\left(\;\frac{\mu }{Y_{X/S}\left(Si-S\right)}\;\right)V_{0}X_{0}\exp\left(\;\mu t\;\right) \end{array}$$

in which *S* and* Si* are, respectively, the crystallized sugar concentration in the medium at the beginning of the feed (g/l) and in the feed solution (g/l), *t* is culture time (h), *V*_0_ is initial culture volume (L), *X* and *X*_0_ are, respectively, the cell concentration at the start of feeding (g/l) and initial cell concentration (g/l), *Y*_*X*/*S*_ is cell yield on sucrose (g of cells/g of crystallized sugar), and *µ* is the specific cell growth rate (h^−1^). Feeding was begun after 18 hours and lasted for 10 hours.

### 2.5. Analytical Techniques

The fermented broth was used for the determination of D(−) lactic acid and residual sucrose. The samples were centrifuged at 7000 ×g for 15 minutes. The supernatant was filtered through a 0.22 *μ*m membrane and used for analysis. The concentrations were determined using high-performance liquid chromatography (HPLC) equipped with a Rezex ROA column (Phenomenex, USA) and a differential refracting index detector (RID-A, Shimadzu). The mobile phase (0.005 M H_2_SO_4_) was fed at a flow rate of 0.6 mL/min and temperature was maintained at 65°C. After the medium optimization process, the optical purity of the lactic acid produced was determined by HPLC using a Chirex 3126 phenomenex column with 1 mM of CuSO_4_ as the mobile phase at 1 mL/min (26°C). Cell growth was determined based on turbidity at 600 nm. The absorbance of the sample measured in a spectrophotometer was correlated to the dry weight mass.

## 3. Results and Discussion

### 3.1. Influence of Concentration of Medium Components on Lactic Acid Production Using Response Surface Methodology

To obtain greater lactic acid production, the culture conditions were optimized using a factorial design. The independent variables selected for the study were crystallized sugar, yeast extract, and GYP salt solution distributed on two levels, two axial points, and three central points. The results obtained after fermentation were expressed as g/l for the amount of lactic acid produced and residual sugar, g/g for yield, and g/L·h for productivity (Table 2). After 48 hours of fermentation, the greatest production was 88.24 g/L of D(−) lactic acid (run 10). In this run, residual sucrose was 25.49 g/L, productivity was 1.84 g/L·h, and the yield was 0.93 g/g.

**Figure 1:**
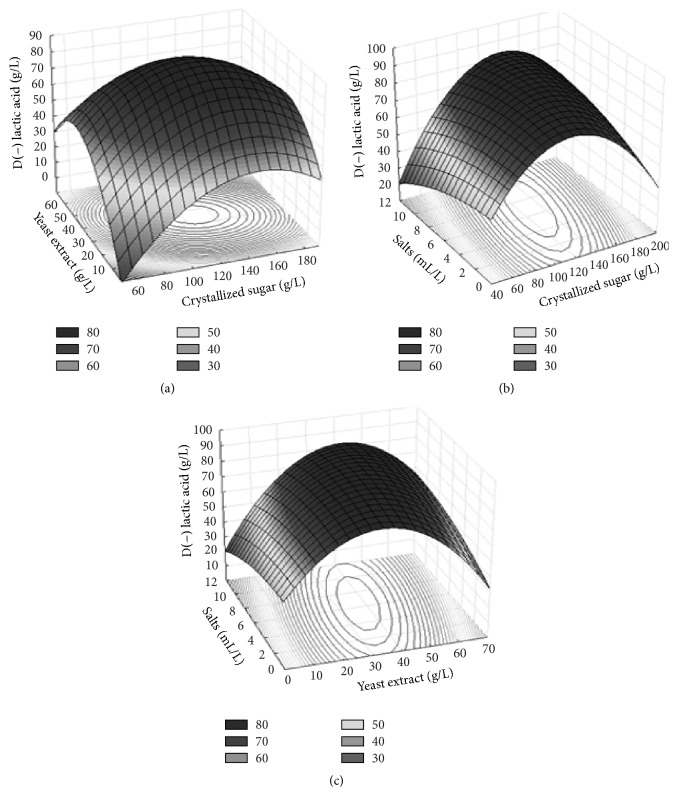
Response surface for lactic acid production by* S. nakayamae*. Interactions between (a) yeast extract and crystallized sugar, (b) salts and crystallized sugar, and (c) yeast extract and salts.

Table 3 displays the results of the analysis of variance (ANOVA) for the selected quadratic model. The coefficient of determination (*R*^2^ = 0.84) suggests that the model explains 84% of the total variation in the response. Fisher's test demonstrated that the model was significant [*F*calc (4.39) > Ft (3.68)] and has a very low probability value (*P*model = 0.03). Among the model terms, interactions *X*_1_*X*_1_ and *X*_2_*X*_2_ were significant with a 95% probability, demonstrating a negative effect, likely due to catabolite repression by the substrates. The results were submitted to multiple regression analysis methods and yielded the regression equation C:

(C) Y=85.34+3.93X1−9.62X1X1+3.44X2−13.78X2X2−0.02X3−2.88X3X3−4,89X1X2+4,89X1X3+5.01X2X3

in which *Y* is the predicted response (D(−) lactic acid production) and *X*_1_, *X*_2_, *X*_3_ are, respectively, the coded values of the test variables crystallized sugar, yeast extract, and salt solution. The response for the regression equation is plotted in Figure 1. The graphs shows the interaction of the variables and optimum levels for D(−) lactic acid production.

Based on the response surface graphs, the medium components that induced the greatest D(−) lactic acid production were 120 g/L of crystallized sugar, 35 g/L of yeast extract, and 7.5 mL/L of salt solution. The concentration of crystallized sugar was selected considering residual sucrose, which should not exceed 10 g/L. The validation of the model for lactic acid production optimization was performed using the selected concentrations in five replicates and the results were expressed as mean values. Under these conditions, production was 89.69 g/L of D(−) lactic acid, productivity was 1.87 g/Lh, the yield was 0.96 g/g, and residual sucrose was 26.67 g/L. These results confirm the validity and usefulness of the model equation. The optimized culture medium was analyzed using HPLC with a chiral column to determine the optical purity of the lactic acid produced. This analysis revealed 98.97% D(−) lactic acid and 1.03% L(+) lactic acid, thereby demonstrating the high optical purity characteristic of* S. nakayamae*.

### 3.2. Effects of Neutralizing Agents on D(−) Lactic Acid Production

A drop in pH occurs during lactic acid fermentation and the microorganism is unable to continue the fermentation. Thus, neutralization is essential and several bases can be used for this purpose, which is closely related to downstream processing [22]. The effect of different neutralizing agents (KOH 10 N, Ca(OH)_2_ 6 N, NaOH 10 N, 5% CaCO_3_, 6% CaCO_3_, and 27% NH_4_OH) on lactic acid production was investigated. Fermentation was conducted using the optimized culture medium at 35°C and pH 6.0 controlled by neutralizing agents automatically added to the process, except CaCO_3_, which was added to the fermentation vessel at the beginning of the process. Fermentation was conducted for 48 hours.

Table 4 displays the results of the pH neutralizing tests. Based on these data, NaOH was selected as the neutralizing agent considering the practicality of the process and the maximum volumetric lactic acid production (103.71 g/L), productivity (2.16 g/L·h), and yield (0.94 g/g). No residual sugar was found in this experiment, which is interesting from the standpoint of further lactic acid purification and polymerization. Moreover, NaOH used as a pH controller in fermentation processes does not generate precipitated waste, making it environmentally friendly [23]. In contrast, the ammoniacal solution was not an appropriate neutralizing agent, as demonstrated by the low lactic acid production (16.05 g/L). This result is in agreement with data reported by other authors studying lactic acid production by* Rhizopus oryzae*, with 24.9 g/L of lactic acid produced when an ammoniacal solution was used as neutralizing agent, as a high concentration of ammonia can be toxic to microbial cells [24].

Calcium carbonate (CaCO_3_) is often used as such a pH controlling agent [25] and yielded the second highest D(−) lactic acid production (91.17 g/L) when used at a proportion of 5% in the present study. In contrast, the 6% proportion inhibited microorganism growth. The low solubility of CaCO_3_ in the fermentation broth could cause problems in the subsequent purification process, as the most used recovery method consumes lime as well as sulfuric acid and also produces a large quantity of calcium sulfate sludge as solid waste [26].

When Ca(OH)_2_ 6 N was used, 88.95 g/L of D(−) lactic acid was achieved. Although this neutralizing agent is affordable, it is necessary to stir the solution constantly using a magnetic plate/stove during fermentation to prevent clogs in the base feeding tubes, resulting in an added cost to the process. A previous study reports D(−) lactic acid production of 127 g/L using Ca(OH)_2_ as the neutralizing agent on the laboratory scale; the author states that the lower osmotic pressure derived from this neutralizing base may be partially responsible for the facilitation of D-lactic acid fermentation [27].

**Figure 2:**
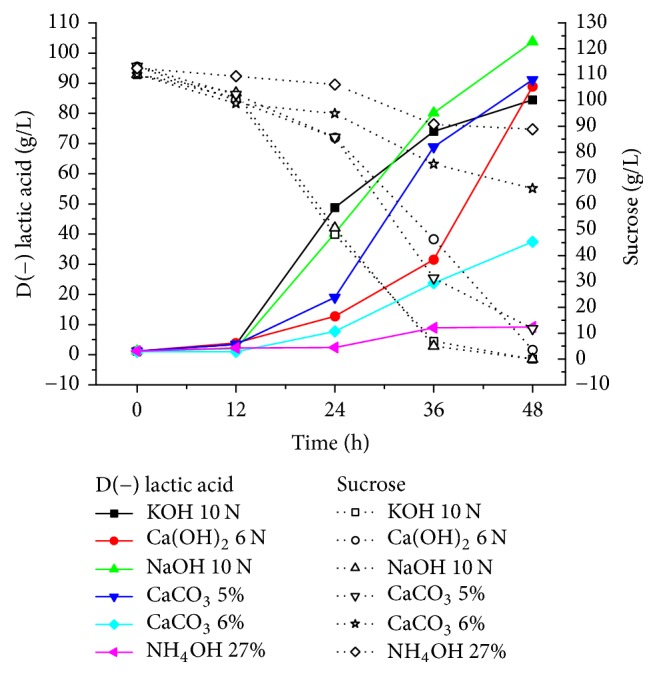
Time course of lactic acid and residual sucrose concentration using different neutralizing agents [KOH, Ca(OH)_2_, NaOH, CaCO, and NH_4_OH]. Culture conditions: GYP modified medium, 35°C pH 6.0, 10% (v/v) inoculums, 100 rpm.

As shown in Figure 2, the process ended without any residual sugar when NaOH or KOH was used as the neutralizing agent. Besides lactic acid production of 84.45 g/L using KOH neutralization, this agent is unaffordable for large scale production. The figure shows the time course of lactic acid and residual sucrose concentration using different neutralizing agents for 48 hours.

### 3.3. Effects of Culture Conditions on Lactic Acid Production

#### 3.3.1. Influence of Temperature and pH on D(−) Lactic Acid Production

**Figure 3:**
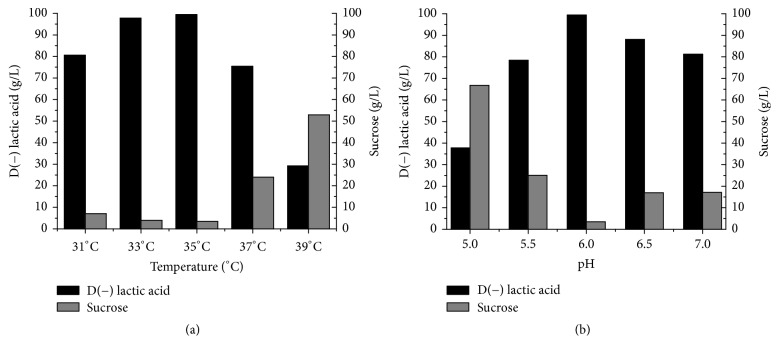
Effect of (a) temperature and (b) pH on D(−) lactic acid production and sucrose consumption by* S. nakayamae*. Culture conditions: GYP modified medium (120 g/L of crystallized sugar, 35 g/L of yeast extract, and 7.5 salt solution) with NaOH 10 N as pH neutralizing agent. (a) GYP modified medium at pH 6.0, under 100 rpm, with 10% inoculum; (b) GYP modified medium at 35°C, under 100 rpm, with 10% inoculum.

The capacity of microorganisms to produce lactic acid is influenced by conditions, such as temperature, pH, agitation, and inoculum percentage, the optimization of which is essential. Temperature and pH are important parameters that affect the fermentation process [28]. D(−) lactic acid produced by the* S. nakayamae* was efficient at pH 6.0, with an optimum temperature at 35°C (Figures 3(a) and 3(b)), reaching a production of 99.43 g/L with a low level of residual sucrose (3.45 g/L). The productivity and yield under these conditions were 2.07 g/L·h and 0.91 g/g, respectively (data not shown). Other authors report achieving a high D-lactate concentration using strains of* Sporolactobacillus* sp. at 42°C [29, 30]. Temperature exerts an influence on the activity of metabolic cells. Most bacteria that convert sugar into lactic acid are classified as either thermophilic or mesophilic, with optimum growth between 20 and 40°C [31].

The optimum pH for lactic acid production by microorganisms ranges from 5.0 to 7.0 and is dependent on the species of microorganism [32]. In the present study, lactic acid production decreased significantly when the pH was lower than 6.0 (Figure 3(b)). Likewise, previous authors report 52.37 g/L of D(−) lactic acid by* Lactobacillus* sp. LMI8 when fermentation was conducted at pH 6.0 [33].

#### 3.3.2. Effect of Agitation and Inoculum Size on D(−) Lactic Acid Fermentation

**Figure 4:**
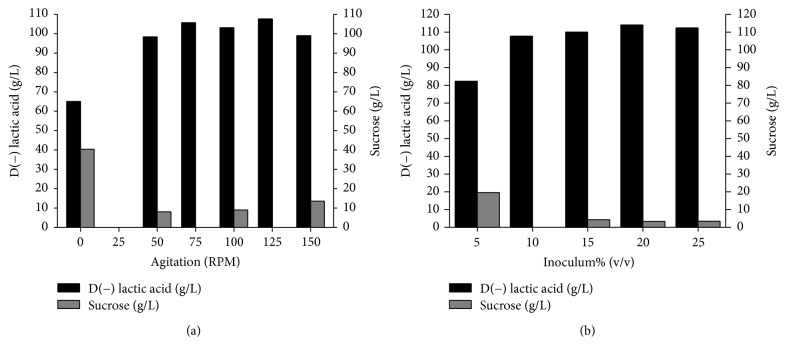
Effect of (a) agitation and (b) inoculum size on D(−) lactic acid production and sucrose consumption by* S. nakayamae*. Culture conditions: GYP modified medium (120 g/L crystallized sugar, 35 g/L yeast extract, and 7.5 mL/L salt solution) with NaOH 10 N as pH neutralizing agent. (a) GYP modified medium at 35°C, pH 6.0, with 10% inoculum; (b) GYP modified medium at 35°C and pH 6.0 under 125 rpm.

The influence of agitation and inoculum size on D(−) lactic acid production was investigated, the results of which are shown in Figure 4. High D(−) lactic acid production (107.67 g/L) was found when agitation was 125 rpm. Moreover, high productivity (2.24 g/Lh) and yield (0.98 g/g) (data not shown) were achieved and no residual sucrose was found under this condition. Stationary conditions were inefficient for lactic acid production (65.03 g/L), possibly due to insufficient homogenization of the culture medium. In contrast, a previous study reported no difference in lactic acid production by* Lactobacillus casei* under stationary conditions or agitation at 100 rpm [34].

Maximum lactic acid production (113.73 g/L) was obtained with low residual sucrose (3.37 g/L) when using 20% (v/v) of inoculum in the culture medium. Moreover, productivity was 2.37 g/Lh and yield was 0.98 g/g under this condition (data not shown). The low lactic acid production (82.34 g/L) with 5% (v/v) inoculum could be attributed to the low density of the starter culture. In a previous study addressing the influence of inoculum size on L(+) lactic acid production by* Rhizopus oryzae* ASC081, production decreased when the inoculum size was less than 10% due to an inadequate enzymatic efficiency [35].

### 3.4. Fed-Batch Fermentation Strategies

**Figure 5:**
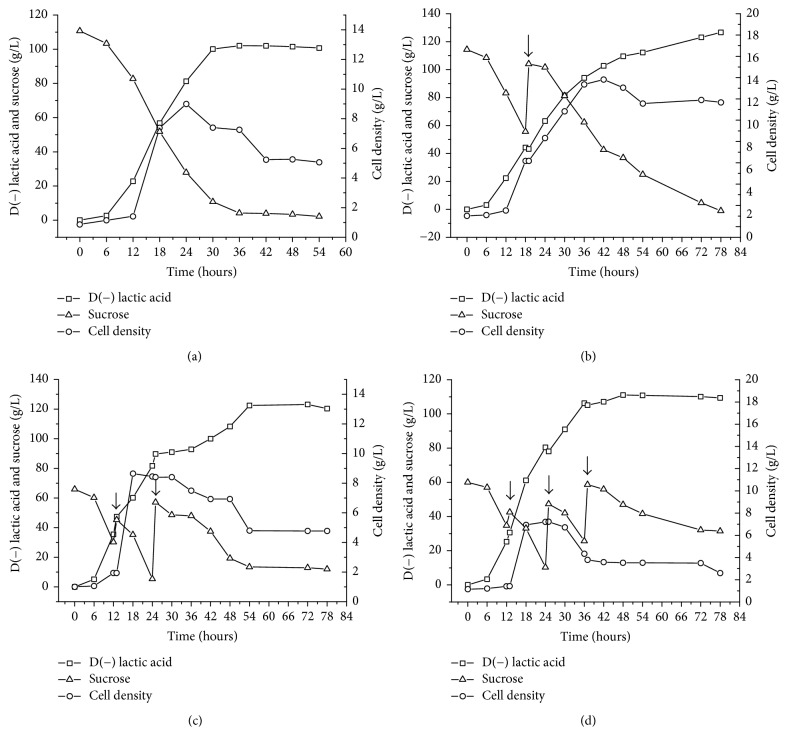
Time courses of sucrose consumption, D(−) lactic acid production, and cell growth in batch fermentation (a), pulse fed-batch (b), and multipulse fed-batch fermentation (c and d) by* S. nakayamae*. Experimental conditions: medium containing 35 g/L of yeast extract and 7.5 mL/L of GYP salts and crystallized sugar (as required); pH 6.0, NaOH 10 N as controlling agent, temperature at 35°C, 125 rpm, and 20% of inoculum (v/v).

Fed-batch cultures were developed to maximize the production and productivity of D(−) lactic acid by* S. nakayamae*. The time courses of cell growth, sucrose consumption, and D(−) lactic acid production during the batch and fed-batch cultures (pulse and multipulse) are shown in Figure 5.

Maximum D(−) lactic acid production (126.64 g/L) was obtained at the end of the single-pulse fed-batch (Figure 5(b)). The highest cell density (11.67 g/L) was also found in this culture. However, it took 78 hours to achieve these results, with productivity of 1.62 g/L·h. Using pulse fed-batch to improve D(−) lactic acid productivity by* Sporolactobacillus inulinus* YBS1-5, Bai et al. (2016) [36] achieved 107 g/L of lactic acid and productivity of 1.19 g/L·h at 90 h.

The fed-batch fermentation with two pulses (Figure 5(c)) initiated at low levels of sucrose demonstrated promising results, achieving the second greatest lactic acid production (122.64 g/L) at 54 hours; at this time, the residual sugar concentration was 13.47 g/L. Moreover, this process had the highest productivity at 13 hours (3.65 g/L·h) in comparison to the other methods. Bai et al. (2003) [37] report similar results studying fed-batch fermentation for L(+) lactic production by* Lactobacillus lactis*.

The multipulse, fed-batch culture with three pulses (Figure 5(d)) was not satisfactory. The amount of sugar added likely caused high osmotic pressure in the cells, resulting in plasmolysis and decreasing both the fermentation rate and sugar utilization. This finding is in agreement with data described by Kotzamanidis et al. (2002) [38]. Other authors report greater production by* Sporolactobacillus* sp. CASD using a multipulse strategy in comparison to a single pulse [30].

**Figure 6:**
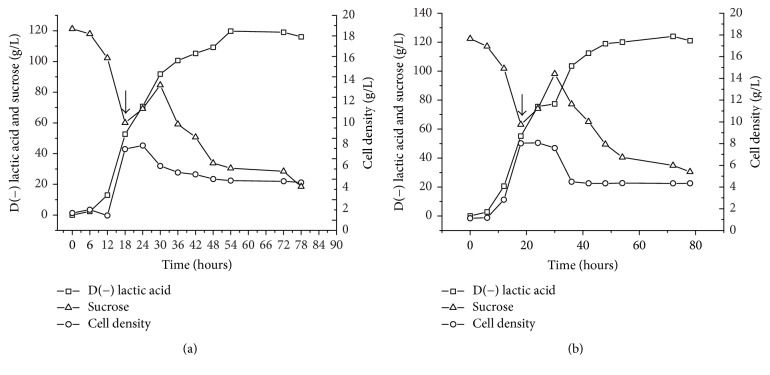
Time courses of sucrose consumption, D(−) lactic acid production, and cell growth in constant feed rate (a) and exponential fed-batch fermentation (b) by* S. nakayamae*. Experimental conditions: medium containing 35 g/L of yeast extract and 7.5 mL/L of GYP salts and crystallized sugar (as required); pH 6.0, NaOH 10 N as controlling agent, temperature at 35°C, 125 rpm, and 20% of inoculum (v/v).

The final D(−) lactic acid concentration with the constant feed rate and exponential fed-batch systems was 119.03 g/L and 121.02 g/L, respectively (Figures 6(a) and 6(b)). These strategies were not satisfactory, as the sucrose concentration was greater than 20 g/L at the end of the process. Although productivity values were low at the end of the process, the highest productivity was 3.15 g/L·h with the exponential fed-batch method at 24 hrs and 3.06 g/L·h with the constant feed rate at 30 hrs. Bernardo et al. (2016) [39] report similar results using a constant feed rate for L(+) lactic acid production by* Lactobacillus rhamnosus* B103. The authors report a slight increase in lactic acid production, but a high residual substrate concentration at the end of the process. Other authors report a 56.5% improvement in L(+) lactic acid production using exponential feeding compared to traditional batch culture, achieving 157.5 g/L of L(+) lactic acid and productivity of 1.88 g/L·h by* Lactobacillus casei* after 84 h of fermentation [40].

Except for the three-pulse feeding strategy, all other fed-batch strategies were able to increase the production and productivity of D(−) lactic acid when compared to batch fermentation (Figure 5(a)), which achieved maximum production and productivity of 106.95 g/L and 2.67 g/L·h, respectively.

The fed-batch process could increase the total substrate content in the bioreactor by maintaining a low substrate concentration during fermentation and avoiding the inhibitory effects of sugar on lactic acid production as well as reducing the negative effects of osmotic pressure on bacterial cells. Other advantages of this process are higher cell concentration and productivity as well as a high titer of lactic acid production at the end of the fermentation process. The final product was not an inhibitory factor, as the lactic acid was presented as a salt (sodium lactate) when NaOH was used as the neutralizing agent. However, the osmotic pressure may be high at the end of the process, which can lead to a reduction in lactic acid production. This decline also could be related to decreasing metabolic potential of an aging microbial biocatalyst, as reported before by Ou et al., 2011 [41]. This paper is a part of a doctoral thesis [41].

## 4. Conclusion

D(−) lactic acid was successfully produced by* S. nakayamae* using fermentation under optimized conditions. The experimental design was very useful for determining the optimal concentrations of constituents that have significant effects on D(−) lactic acid production. This study has shown that NaOH is an effective, environmentally friendly pH controlling agent. Moreover, pH, temperature, agitation, and inoculum size all exerted a significant influence on the production process. The fed-batch process was able to increase both the production and productivity of D(−) lactic acid. These findings demonstrate that* S. nakayamae* is a promising strain for D(−) lactic acid production.

## Figures and Tables

**Table 1:** Variables and respective levels for D(−) lactic acid production using response surface methodology.

Variable	Levels					
		−1.68	−1	0	1	1.68
Crystallized sugar (g/L)	*X*_1_	52.8	80	120	160	187.2
Yeast extract (g/L)	*X*_2_	1.4	15	35	55	68.6
GYP salt solution (ml/L)	*X*_3_	0.9	3	6	9	11.04

**Table 2:** RSM design with real values and experimental results for lactic acid production, productivity, yield, and residual sugar concentration.

Run	Experimental factors			Response variable			
	Crystallized sugar (g/L)	Yeast extract (g/L)	Salt solution (mL/L)	Lactic acid	Productivity	*Y*_p/s_	Residual sugar
	*X*_1_	*X*_2_	*X*_3_	g/L	g/Lh	g/g	g/L
1	80.0	15.0	3.0	65.13	1.36	0.81	0
2	160.0	15.0	3.0	59.02	1.23	0.75	81.41
3	80.0	55.0	3.0	61.32	1.28	0.97	17.00
4	160.0	55.0	3.0	58.36	1.22	0.88	93.34
5	80.0	15.0	9.0	26.64	0.56	0.97	52.44
6	160.0	15.0	9.0	62.84	1.31	0.84	85.00
7	80.0	55.0	9.0	65.59	1.37	0.99	14.03
8	160.0	55.0	9.0	59.51	1.24	0.82	87.35
9	120.0	35.0	0.9	71.01	1.48	0.98	47.20
10	120.0	35.0	11.0	88.24	1.84	0.93	25.49
11	120.0	1.40	6.0	44.10	0.92	0.98	74.86
12	120.0	68.5	6.0	53.53	1.12	0.99	65.84
13	52.8	35.0	6.0	50.86	1.06	0.96	0.00
14	187.2	35.0	6.0	70.31	1.46	0.77	96.34
15	120.0	35.0	6.0	87.98	1.83	0.98	30.64
16	120.0	35.0	6.0	81.85	1.71	0.94	33.34
17	120.0	35.0	6.0	85.33	1.78	0.89	24.35

*X*_1_  = crystallized sugar; *X*_2_  = yeast extract; *X*_3_  = salt solution. Culture conditions: 35°C, initial pH at 6.0 neutralized by 5% CaCO_3_, stationary fermentation, and 10% inoculum. Fermentation was conducted for 48 hours.

**Table 3:** ANOVA with estimated regression coefficients for D(−) lactic acid production.

Source	Coefficient	Sum of squares	Degrees of freedom	Mean square	*F* ratio	*P*
*X*_1_	3.93	211.632	1	211.632	2.351	0.169
*X*_1_ *X*_1_	−9.62	1043.505	1	1043.505	11.594	0.011^*∗*^
*X*_2_	3.44	161.814	1	161.814	1.798	0.221
*X*_2_ *X*_2_	−13.78	2141.408	1	2141.408	23.794	0.001^*∗*^
*X*_3_	−0.02	0.005	1	0.005	0.000	0.994
*X*_3_ *X*_3_	−2.88	94.113	1	94.113	1.045	0.340
*X*_1_ *X*_2_	−4.89	191.395	1	191.395	2.126	0.188
*X*_1_ *X*_3_	4.89	191.982	1	191.982	2.133	0.187
*X*_2_ *X*_3_	5.01	200.901	1	200.901	2.232	0.178
Model	—	3557.58	9	395.28	4.39	0.03
Error	—	629.97	7	89.99	—	—
Lack of fit	—	611.07	5	122.21	12.93	0.07
Pure error	—	18.90	2	9.45	—	—
Total	—	4187.55	16	—	—	—

*X*_1_  = crystallized sugar; *X*_2_  = yeast extract; *X*_3_  = salt solution. ^*∗*^Statistically significant at 95% probability level.

**Table 4:** Comparison of D-lactic acid fermentation by *S. nakayamae* using different neutralizing agents.

Neutralizing agent	Price	Used in fermentation	Cost in fermentation	D(−) lactic acid^*∗*^	*P*	*Y*_p/s_	RS^*∗*^
	R$/kg	g/300 mL	R$	g/L	g/L·h	g/g	g/L
CaCO_3_ 5%	44.00	15.00	0.66	91.17	1.90	0.90	11.76
Ca(OH)_2_	40.00	11.11	0.44	88.95	1.85	0.81	3.52
NaOH	51.00	12.00	0.61	103.71	2.16	0.94	0.00
KOH	86.00	5.61	0.48	84.45	1.98	0.79	0.00
NH_4_OH	17.20	9.17	0.17	16.05	0.33	0.13	88.85
CaCO_3_ 6%	44.00	18.00	0.79	37.46	0.78	0.85	65.99

*P*  = productivity; *Y*_p/s_  = Yield; RS = residual sucrose. ^*∗*^Normalized lactic acid and sucrose titer was calculated from measured titer (not shown) from fermentation broth with dilution ratio of neutralizing agent used. Productivity and yield were calculated considering normalized data.
